# Optimizing surgical decision-making for lung adenocarcinoma by preoperatively identifying pathological high-risk factors: retrospective development and prospective validation of a deep learning model

**DOI:** 10.1097/JS9.0000000000005084

**Published:** 2026-03-17

**Authors:** Xincheng Li, Jianhua Liu, Xiangyu Meng, Jun Lu, Wei Feng, Jing Zhou, Bin Hu, Xiumei Hu, Ying Ji

**Affiliations:** a Department of Thoracic Surgery, Beijing Institute of Respiratory Medicine and Beijing Chao-Yang Hospital, Capital Medical University, Beijing, China; b Center for Applied Statistics, School of Statistics, Renmin University of China, Beijing, China; c Department of Pathology, Beijing Chao-Yang Hospital, Capital Medical University, Beijing, China; d Department of Cardiothoracic Surgery, The Third Xiangya Hospital of Central South University, Changsha, China

**Keywords:** deep learning, intraoperative FS, lung adenocarcinoma, pathological high-risk factors, surgical decision-making

## Abstract

**Background:**

Accurate identification of pathological high-risk factors (PHRFs) in early-stage lung adenocarcinoma (LUAD) is critical for optimizing surgical decision-making. However, reliance on intraoperative frozen section (FS) assessment is limited by insufficient sensitivity. This study aimed to retrospectively develop and prospectively validate a deep learning model (a knowledge-based graph convolutional network, KB-GCN) based on preoperative CT scans to identify PHRFs in LUAD.

**Methods:**

We retrospectively developed and externally validated a KB-GCN using two cohorts (A: 268 patients/297 lesions for training and internal validation; B: 68 patients/75 lesions for external validation). We then conducted a pre-registered, single-center, prospective observational validation in 200 consecutive surgical candidates with early-stage LUAD. Before prospective enrollment, the model architecture, weights, preprocessing pipeline, and the decision threshold (0.40, determined from the retrospective phase) were locked. For each patient, a preoperative prediction was generated before intraoperative FS and final pathology (FP); the clinical team was blinded to the model output. The performance of the locked model and FS was compared with FP.

**Results:**

In retrospective validation, the KB-GCN model achieved an area under the curve (AUC) of 0.92 (95% CI: 0.86–0.97) in the internal validation cohort and 0.88 (95% CI: 0.81–0.94) in the external validation cohort. The KB-GCN model outperformed all 6 compared classical 2D/3D CNN models (best comparative AUC: 0.79). In prospective validation, intraoperative FS achieved an overall sensitivity of 59% and accuracy of 77% for detecting PHRFs, misclassifying 40.6% (39/96) of PHRF-positive cases. In contrast, the KB-GCN model demonstrated significantly higher overall sensitivity (82%) and AUC (0.83), although with lower specificity (75% vs FS: 92%). The KB-GCN model showed superior performance in part-solid nodules (PSN, AUC: 0.86) and in 2–3 cm tumors (AUC: 0.86), with moderate performance in ≤1 cm tumors (AUC: 0.82) and in 1–2 cm tumors (AUC: 0.79).

**Conclusion:**

This study retrospectively developed and, for the first time, prospectively demonstrates that a deep learning model based on preoperative chest CT predicts PHRFs, achieving significantly higher sensitivity for identifying PHRFs in early-stage invasive LUAD than conventional intraoperative FS. Despite slightly lower specificity, the KB-GCN model effectively compensates for the critical sensitivity deficit of FS, particularly for tumors (1–3 cm) containing solid components. Preoperative deep learning assessment combined with intraoperative FS provides thoracic surgeons with more comprehensive and accurate information to optimize surgical decisions (e.g., extent of resection: lobectomy or sublobar resection). Future development requires integrating this preoperative model with intraoperative FS assessment into standardized workflows.

## Introduction

Lung cancer has always been among the most frequently diagnosed cancers threatening people’s health worldwide^[^1,2^]^. Especially with the popularization of low-dose computed tomography (CT) screening technology, more and more early-stage lung cancer has been detected^[^3^]^. For specific patients with early-stage lung cancer, sublobar resection (wedge resection or segmentectomy) has been demonstrated to achieve a comparable long-term prognosis to lobectomy while preserving more healthy lung tissue, provided adequate margins and lymph node assessment are ensured (as in lepidic-predominant adenocarcinoma patients without high-risk pathological subtypes). Evidence such as the JCOG0802/WJOG4607L trial (2022) has made sublobar resection an increasingly important surgical approach for early-stage lung cancer^[^4^]^.

However, key studies show sublobar resection suitability depends heavily on specific tumor pathological features^[^5^]^. Multiple studies confirm these PHRFs and include: high-grade pathological subtypes (>5% micropapillary, solid, or complex glandular architecture), visceral pleural invasion (VPI), spread through air spaces (STAS), and lymphovascular invasion (LVI)^[^6–10^]^. For early-stage invasive adenocarcinoma, numerous studies indicate that when PHRFs are present, sublobar resection is associated with higher local recurrence. Even when only a small fraction (>5%) of micropapillary or solid components is present, outcomes after sublobar resection are inferior to those after lobectomy^[^11–14^]^. Complex glandular architecture – designated by the International Association for the Study of Lung Cancer (IASLC) as a high-grade component – is associated with adverse prognosis and nodal metastasis^[^15–18^]^. STAS, VPI, and LVI further increase the risk of locoregional failure after sublobar resection^[^19–24^]^. Accordingly, current consensus generally supports lobectomy with systematic lymph node dissection when PHRFs are identified. In contrast, sublobar resection is considered oncologically safe for PHRF-negative LUAD (e.g., lepidic-predominant or carefully selected acinar/papillary subtypes), provided adequate margins (≥2 cm or ≥tumor diameter) and appropriate nodal assessment are achieved^[^25^]^. Therefore, accurately identifying these PHRFs before or during surgery is critical for decision-making. This ensures low-risk patients (LUAD without PHRFs) benefit from sublobar resection, preserving lung function and quality of life. It also prevents high-risk patients from receiving sublobar surgery with insufficient local control. Notably, lobectomy is not universally mandated in every PHRF-positive case; in patients with limited cardiopulmonary reserve, significant comorbidity, or unfavorable anatomy, sublobar resection may be cautiously selected with rigorous intraoperative margin assessment and consideration of adjuvant therapy.

Currently, intraoperative FS assessments are the primary method for real-time pathology assessment to guide the choice between sublobar resection and lobectomy. However, FS has significant limitations in diagnosing key high-risk factors. Its sensitivity for detecting high-grade subtypes (especially micropapillary) is low^[^13,26^]^. For micropapillary-predominant adenocarcinoma, FS specificity is high (99%), but sensitivity is only 21%^[^27^]^. For solid-predominant adenocarcinoma, the FS sensitivity and specificity are 79 and 94%, respectively^[^27^]^. STAS diagnosis sensitivity by FS is only 44–54%, and STAS assessment is also susceptible to artifacts. Accurate VPI diagnosis relies on elastic fiber staining, which is difficult during surgery^[^28,29^]^. Overall, the agreement between FS and FP for major histological subtypes is only about 68%, and agreement between different observers is also low, at around 64%^[^27^]^. Surgeons rely heavily on intraoperative frozen pathology results for surgical decisions, and these FS limitations are problematic: They may lead to misclassifying high-risk patients as suitable for sublobar resection. Alternatively, they can cause prolonged surgical waiting times as surgeons might wait for the pathological assessment of PHRFs. More reliable and efficient preoperative prediction methods are urgently needed.

HIGHLIGHTS

- AI predicts pathological high-risk factors in early lung adenocarcinoma via preoperative CT.
- Superior sensitivity to frozen section (82 vs 59%), reduces missed diagnosis by 40%.
- Prospective AUC: 0.83, external validation AUC: 0.88 with strong generalizability.
- Preoperative AI optimizes surgical decisions by identifying high-risk pathology.

Artificial intelligence (AI) has provided a viable approach for analyzing medical images^[^30–32^]^. The deep learning (DL) models based on convolutional neural network (CNN) has shown promising results in some chest CT image analysis tasks^[^13,30–37^]^, such as pulmonary nodule segmentation^[^34,36,38–40^]^, judgment of benign and malignant nodules^[^40,41–45^]^, and distinguish LUAD from pre-LUAD^[^41–43,46–48^]^. A recent study by Zhou *et al* (2022) demonstrated that the accuracy of the DL algorithm in the prediction of pathological subtypes of LUAD is significantly better than that of radiologists (77.6 vs 66.23%)^[^49^]^. Studies have shown that AI technology can also predict tumor gene mutations and chemotherapy sensitivity from digital pathology images. There are specific differences in the manifestations of different pathological subtypes of lung adenocarcinoma, so we can use DL models to identify PHRFs in lung adenocarcinoma. This study adheres to the TITAN Guidelines 2025 for governing AI in surgical research^[^50^]^.

**Figure 1.**
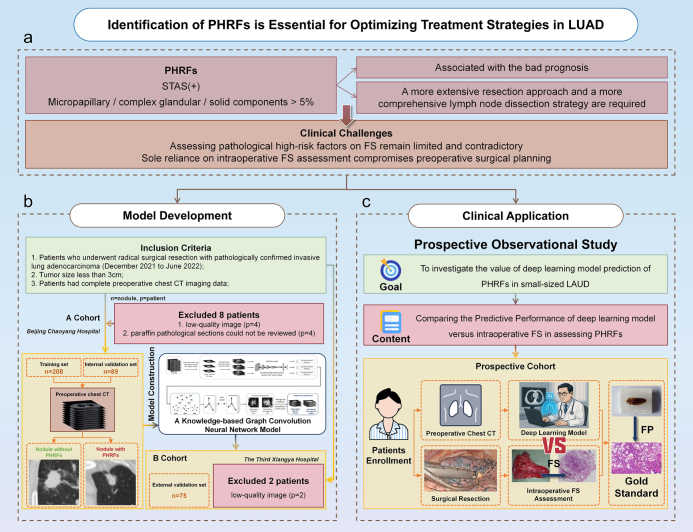
Overall flowchart in this study. (A) Clinical significance and challenges: Highlighting the importance of identifying PHRFs and limitations of FS evaluation in preoperative planning. (B) Model development and validation: KB-GCN model was trained and optimized using a training cohort, internally and externally validated with an internal validation cohort, and an external validation cohort. Performance was further validated in a prospective observational cohort. (C) Clinical application: Comparison of the KB-GCN model and intraoperative FS assessment for PHRFs evaluation. The workflow includes preoperative CT–based AI prediction, intraoperative FS analysis, and final pathological confirmation to guide surgical decision-making.

In this work, we conducted a two-phase study. First, we retrospectively developed and externally validated a knowledge-based graph convolutional neural network (KB-GCN model) using thin-slice preoperative CT to predict PHRFs (>5% high-grade components and STAS) in early-stage invasive LUAD. Second, we performed a pre-registered, single-center, prospective observational validation in which a locked version of the model (architecture, weights, preprocessing, and a prespecified decision threshold of 0.40) was applied preoperatively to consecutively enrolled surgical candidates. For each case, predictions were generated before intraoperative FS and FP, blinded to the clinical team, and evaluated against FS within the intraoperative workflow using FP as the reference standard. This design enables rigorous assessment of actual prospective performance while preserving the practical clinical context in which such a tool would be applied. The goal is to prove that the deep learning model can effectively predict PHRFs in early-stage LUAD preoperatively. This will provide surgeons with preoperative decision support. Based on the prediction results, surgeons can plan the surgical approach (lobectomy or sublobar resection). This shifts the decision point earlier preoperatively, overcoming the limitations of intraoperative FS assessment. The overall research approach and framework are presented systematically in Figure 1. This cohort study has been reported in line with the STROCSS guidelines^[^51^]^.

## Materials and methods

### Development cohorts

This study retrospectively analyzed 346 consecutive subjects of pT1 lung adenocarcinoma (the 8th edition lung cancer staging criteria of the IASLC) who underwent surgical resection in two medical centers (Beijing Chao-Yang Hospital and the Third Xiangya Hospital) from December 2021 to June 2022. Following the exclusion criteria (Excluded 6 cases with low-quality chest CT images and 4 cases in which diagnoses based on permanent sections could not be reviewed), a total of 372 pathologically confirmed pulmonary nodules from 336 subjects (137 men, 199 women) were used to develop and validate the proposed DL model. Supplemental Digital Content Part I, available at: http://links.lww.com/JS9/H79, shows the detailed inclusion/exclusion criteria for the registered patients in this retrospective study from the two centers.

All patients were pathologically confirmed to have invasive LUAD. All the patients from the two centers received a thin-slice CT scan with slice thickness ranging from 0.5 mm to 1.5 mm, with an average of 0.625 mm. No significant differences were observed between the two centers (*P* > 0.05).

### Prospective observational cohort study

The prospective observational cohort (Prospective Cohort) enrolled at Beijing Chao-Yang Hospital (January 12–1 September 2023) was designed to validate the diagnostic concordance between our KB-GCN model and intraoperative FS analysis for identifying PHRFs in early-stage lung adenocarcinoma. The study was registered in the Chinese Clinical Trial Registry (identifier: ChiCTR2300073455; https://www.chictr.org.cn). Supplemental Digital Content Part II, available at: http://links.lww.com/JS9/H79, shows the detailed inclusion/exclusion criteria for the registered patients in this prospective study. While VPI and LVI are recognized as high-risk features, their reliable intraoperative assessment by FS remains limited. STAS has low sensitivity and susceptibility to artifacts, but can be attempted on FS. To ensure a rigorous and executable prospective comparison that reflects real-world intraoperative workflows, we defined the primary endpoint as identifying PHRFs that can be reliably assessed by FS – specifically, >5% high-grade components and STAS.

**Figure 2.**
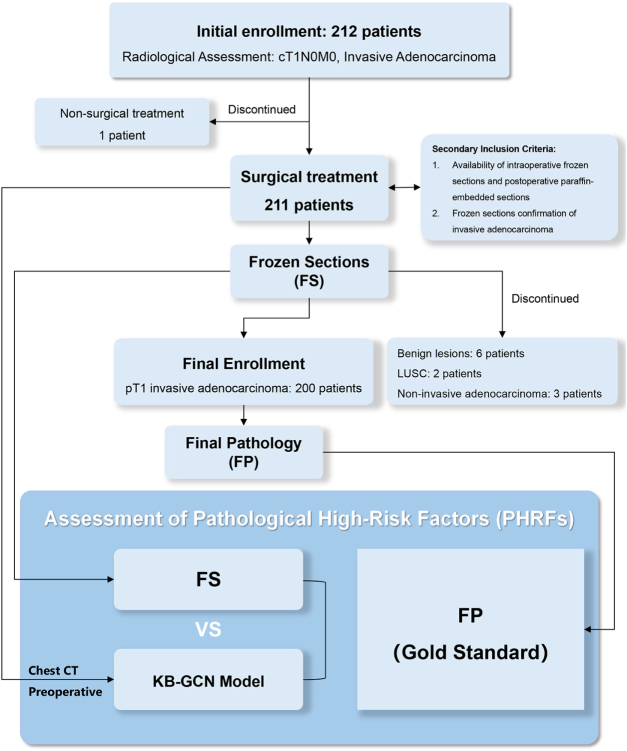
Overall flowchart in the prospective observational cohort study. Flowchart of the Prospective Cohort study and subject enrollment process. Initially, 212 patients with radiological diagnosis of cT1N0M0 invasive adenocarcinoma were enrolled, with 200 early-stage invasive lung adenocarcinoma patients ultimately included. FP served as the gold standard for evaluating PHRFs. The KB-GCN model compared the presence of PHRFs through preoperative CT and FP.

A total of 212 patients underwent preliminary screening. Among these, one patient was excluded for non-surgical treatment, six for benign lesions, two for squamous cell carcinoma (LUSC) confirmed postoperatively, and three for non-invasive adenocarcinoma. No patient was excluded because of inadequate FS or CT artifact. The final analysis, therefore, included 200 eligible patients with both evaluable FS and diagnostic-quality preoperative CT scans. Consequently, 200 patients were ultimately included in the final analysis with an average age of 61.6 years. The cohort comprised 68.5% females (137/200) and 31.5% males (63/200). The Eastern Cooperative Oncology Group (ECOG) performance status of all patients matched the inclusion criteria, with all participants having a score of 0 or 1. For example, 84% of patients (168/200) had a score of 0, reflecting a fully active status. In comparison, 16% (32/200) had a score of 1, indicating some limitations in physical activity but no need for assistance in daily living. Figure 2 presents the flowchart in the Prospective Observational Cohort Study.

Before enrolling the first prospective patient, we froze the model architecture, weights, image preprocessing pipeline, and the decision threshold (0.40, derived from the retrospective phase). For each prospective case, the KB-GCN model prediction was generated before surgery and before FS/FP results were available. The surgical and pathology teams were blinded to the AI output, and AI predictions did not influence intraoperative decisions. No model retraining, recalibration, or threshold adjustment was performed using prospective data.

### Sample size estimation

The sample size for the primary endpoint (sensitivity difference between the KB-GCN model and FS) was calculated using McNemar’s test for paired proportions, restricted to PHRF-positive cases. Let ${\text{p}}_{\text{10}}$ and ${\text{p}}_{\text{01}}$ denote the proportions of discordant pairs (KB-GCN+/FS− and KB-GCN−/FS+, respectively), with $\Delta {\text{=p}}_{\text{10}}{\text{-p}}_{\text{01}}$ (expected sensitivity difference) and ${\text{q=p}}_{\text{10}}{\text{+p}}_{\text{01}}$ (discordant rate). For a two-sided α = 0.05 and power ≥ 0.80 $({\text{z}}_{\text{1-}\alpha \text{/2}}\text{=1}.\text{96}$, ${\text{z}}_{\text{1-}\beta }\text{=0}.\text{84})$, the required number of PHRF-positive cases m was:

$$\text{m=}\frac{{\left({\text{z}}_{\text{1-}\alpha \text{/2}}{\text{+z}}_{\text{1-}\beta }\right)}^{\text{2}}\cdot \text{q}}{\Delta^{\text{2}}}\text{=}\frac{\text{7}.\text{84q}}{\Delta^{\text{2}}}$$.

Based on prior data, we assumed Δ=0.18--0.20 and q=0.30--0.35, yielding m=59--85. With an anticipated PHRF-positive prevalence $\left(\pi_{\text{obs}}\right)$ of 50%, the total required sample size was $\text{N=m/}\pi_{\text{obs}}\text{=118--170}$. To ensure robustness, we enrolled 212 patients, of whom 200 met the inclusion criteria (including 96 PHRF-positive cases), exceeding the calculated requirement.

### Image preprocessing

To address variations in CT scanners and acquisition settings (e.g., slice thickness ranging from 0.5 mm to 1.5 mm, with an average of 0.625 mm), we implemented several standardization steps. First, all CT images were resampled to a uniform voxel spacing of 1 mm × 1 mm × 1 mm using linear interpolation based on the scan intervals stored in the metadata, ensuring consistent spatial resolution. Second, the Hounsfield unit (HU) values were truncated to the range [−1200, 600] to focus on the relevant lung imaging range, and then normalized to [0, 255] for computational efficiency. These preprocessing steps minimized the impact of scanner-specific differences and acquisition parameters, enhancing the model’s robustness.

### Nodule annotations

The nodules from the two centers were labeled by reviewing the CT images prior to surgery. For each medical center, the annotation procedure was performed by two experienced thoracic radiologists (with>8 years of experience reading lung CT) and one thoracic surgeon (with >10 years of experience reading lung CT) in a two-stage process. In the first stage, each radiologist or thoracic surgeon independently annotated the centroid coordinates (e.g., *X, Y*, and *Z*) and the maximum diameter of each pulmonary nodule by carefully reading the preoperative CT images. It should be noted that this marking method has been widely used in the well-known public LIDC-IDRI dataset^[^52^]^. In the second stage, each doctor independently reviewed the labels of the other doctors and made minor adjustments to their own coordinates. The final coordinates (i.e., *X, Y*, and *Z*) of each pulmonary nodule were determined by calculating the median value of all the annotated coordinates.

### Histologic evaluation

To support time-sensitive intraoperative decision-making while preserving the rigor required for final histologic adjudication, we implemented a standardized pathology workflow spanning intraoperative FS and permanent sections (PS). The workflow emphasizes targeted sampling at the tumor-normal interface, independent dual-reader assessment with rapid consensus adjudication, and systematic quality control across equipment and slide preparation. All procedures and reporting conventions adhere to IASLC/ATS/ERS and WHO criteria and were designed to dovetail with the study’s high-risk histologic endpoints.

For the FS phase, two senior pathology technologists are dedicated to grossing and rapid cryostat sectioning. Two senior thoracic pathologists independently review the FS slides and document their impressions. When the two diagnoses differ, or when critical determinations that could alter the surgical plan are uncertain – for example, the presence of invasion, whether high-grade components exceed 5% (micropapillary, solid, or complex glandular), or a suspicion of STAS – a third senior thoracic pathologist is engaged immediately. As needed, a brief multi-head microscope conference is convened to achieve consensus within 10–15 minutes, matching intraoperative decision timelines. Communication of the FS result to the surgical team is contemporaneous and includes explicit notation of any diagnostic uncertainty and its potential implications.

FS sampling is performed immediately after resection. Specimens are oriented and sliced along the largest tumor diameter, and blocks are targeted to the tumor-normal interface and to any radiologically or grossly solid components, reflecting common real-world protocols. As a standard approach, 1–2 blocks are submitted; each block is sectioned at 2–3 levels, with focus on the largest-diameter interface. For tumors ≥1 cm, part-solid nodules, or morphologically heterogeneous lesions, two blocks are prioritized and a third block may be added if time permits to mitigate sampling error. Rapid hematoxylin and eosin (H&E) staining is used; no inflation techniques or special stains are performed intraoperatively. FS diagnoses follow IASLC/ATS/ERS categories (AAH/AIS/MIA/invasive adenocarcinoma), and the report annotates confounding factors (e.g., alveolar collapse, interstitial fibrosis, intra-alveolar macrophages) when they could mimic or obscure invasion. When residual uncertainty persists after expedited consensus, a conservative FS diagnosis is issued with recommendations for additional sampling or deferral of definitive grading to PS.

For PS, tissue is fixed in 10% neutral buffered formalin, paraffin-embedded, and serially sectioned at 4–5 μm. At least two blocks covering the entire tumor are processed, with additional levels obtained as needed to resolve areas of uncertainty. Routine H&E is performed for all blocks, and special stains are applied as indicated, including PAS for mucin. Immunohistochemistry (e.g., CK7, TTF-1, napsin A) is performed per institutional practice to support complex differential diagnoses. Invasive tumor size is recorded as the most significant invasive focus; when invasion is multifocal, the aggregate extent is estimated according to IASLC/ATS/ERS/WHO guidance. For invasive adenocarcinoma, histologic subtypes are recorded in 5% increments, and STAS is evaluated on PS as part of final pathologic risk stratification. Two senior thoracic pathologists independently review all PS slides, including special stains. If discrepancies arise in key features – such as invasive size, STAS, or the quantification of high-grade components – a third senior pathologist participates in a multi-head microscope review to reach agreement. Deeper levels and ancillary stains are obtained when necessary. The final sign-out reflects majority agreement or the adjudicating pathologist’s determination and documents that multi-observer review was performed.

The technical platform includes a medical-grade cryostat, a rapid stainer, and a coverslipping station for FS, and research-grade microscopes for individual reviews, alongside a multi-head microscope (≥3 heads) for consensus conferences. Slide quality is monitored against predefined criteria (intact sections, crisp H&E staining, distinct cell borders, clear nuclear-cytoplasmic contrast, minimal ice-crystal or cutting artifacts). Daily quality control includes cryostat calibration, stain quality checks, and spot audits of FS slides, with documentation of any nonconformities and corrective actions.

**Figure 3.**
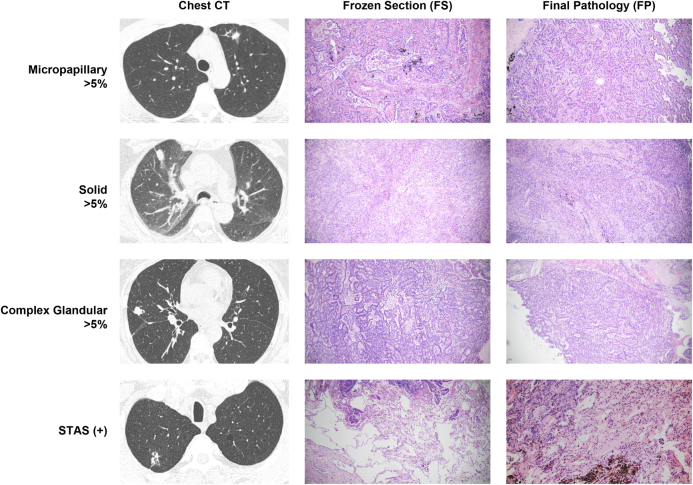
Representative Preoperative CT Scans and Histologic Slides of Different PHRFs. This figure illustrates typical cases of lung adenocarcinoma with distinct PHRFs. Each row displays corresponding preoperative CT images (left column), intraoperative frozen sections (middle column), and postoperative histological sections (right column).

Reader verification and disagreement resolution are embedded in both phases. For FS, concordant dual-reader diagnoses are issued immediately; discordant cases or diagnoses with potential to change surgical management (e.g., MIA vs invasive adenocarcinoma, >5% high-grade components) trigger a rapid three-reader consensus review. The discussion explicitly addresses the solid and micropapillary components, stromal reaction as a surrogate for invasion, and potential pitfalls such as collapse and inflammation. When uncertainty cannot be fully resolved intraoperatively, the FS report remains conservative and transparent, with targeted guidance to the surgical team. For PS, additional levels and special stains are leveraged to adjudicate disagreements, ensuring that the final diagnosis is both comprehensive and reproducible. Typical cases with their CT images and histologic slides were presented in Figure 3.

### Model development and validation

DL models with CNN architectures were widely used for analyzing medical images^[^53–57^]^. Most previous studies captured image features using two-dimensional (2D) or three-dimensional (3D) convolution kernels^[^58–61^]^. In this study, we train a novel DL model with graph convolutional neural network (GCN) to predict the PHRFs of lung adenocarcinoma. Different from previous DL models with 2D or 3D feature extractors, the DL model with GCN structure captured the feature information of each nodule by constructing a spatial relationship graph.

The focus of a GCN model is to establish a graph that contains several nodes and edges between them. In this study, a node represents a slice of a CT, and an edge represents the relationship between slices. To implement the proposed KB-GCN model, we first adopt a pretrained VGG16 model to extract a 512-dimensional feature for each slice of the nodule. Then, based on the natural position of each slice in the nodule, we construct a reticular graph for each nodule. Edges were defined to reflect axial spatial continuity and proximity between slices, so that the GCN could aggregate context across the native slice stack using the pretrained VGG16 features as knowledge-based node attributes. Third, the reticular graphs with 512-dimensional feature vectors as node features were used as input to the GCN model.

Compared with conventional 2D/3D CNN models, this study adopts a graph convolutional network (GCN) framework, termed the KB-GCN model, to model inter-slice relationships within a pulmonary nodule. The “KB” component (knowledge base) refers to the transfer-learning module used for extracting discriminative slice-level features, which subsequently serve as node attributes for graph construction. Specifically, a pretrained VGG16 network was adopted as the backbone and fine-tuned on cropped CT slices. For each case, a 60 × 60 patch centered on the radiologist-annotated nodule center was extracted from each slice, and grayscale images were replicated to form three-channel inputs 60 × 60 × 3. The model was trained using a 70/30 hold-out split with binary cross-entropy loss, the Adam optimizer (learning rate 5 × 10^−5^, and batch sizes of 60 and 40 for the training and test sets, respectively. Ten fine-tuning runs were conducted with different initializations, and the model that achieved the best accuracy was selected. The output of the fully connected layer from this fine-tuned model was used as the slice-level feature vector.

To capture spatial correlations among slices, a fully connected undirected graph was constructed for each nodule, where all slices were treated as nodes linked by bidirectional edges. This design enables each node to aggregate information from all slices while maintaining its own feature specificity. The final GCN model consisted of four layers, organized into two Dropout blocks, each with a dropout rate of 0.5. The model was trained with cross-entropy loss using the Adam optimizer (learning rate 0.01) for up to 400 epochs. The checkpoint achieving the best validation accuracy was retained for testing and performance evaluation. The model was trained and internally validated on the A cohort and externally validated on the B cohort. To predict the probability of PHRFs for a nodule, we average the predicted probabilities of each slice from the GCN model to produce a score between 0 and 1. More details on model implementation are provided in Supplemental Digital Content Figure 1, available at: http://links.lww.com/JS9/H78.

To further demonstrate the effectiveness of the proposed model, we compare it with other state-of-the-art methods. We included a number of benchmark models that used 2D or 3D CNN models. Particularly, we adopted both the 2D and 3D versions of the VGG16, Inception V3^[^62^]^, and ResNet50 models to perform our task. Thereafter, another six models were included for comparison purposes. For more detailed implementations of the comparison studies, details are provided in Supplemental Digital Content Table 1, available at: http://links.lww.com/JS9/H79.

### Visual interpretability

To address model explainability, we employed a feature map visualization approach. Note that the GCN component in our framework was implemented using the tf_geometric package, which encapsulates the internal hierarchical connections. This design prevents direct access to the intermediate feature maps required for generating gradient-weighted class activation maps (Grad-CAM). As a primary alternative to illustrate the model’s focus, we visualized the output feature maps of the first convolutional layer within the transferred convolutional backbone (Supplemental Digital Content Figure 3, available at: http://links.lww.com/JS9/H78). These visualizations demonstrate that the pretrained front-end convolutional layers can be activated by fundamental low-level visual characteristics, such as the morphology and boundary structures of pulmonary nodules. While these early layers capture basic patterns, the subsequent deeper layers in the network are designed to progressively integrate this information to form more abstract and semantically rich representations pertinent to the classification task. This analysis provides valuable insight into the model’s initial feature extraction process.

### Statistical analysis

The performance of the proposed KB-GCN model was comprehensively evaluated with six metrics: AUC, sensitivity, specificity, PPV, NPV, and F1 score. For the GCN-related and 2D CNN models, we first calculate the high-risk factor probability of a slice, and average the probabilities of all slices to the nodule-level probability score from 0 to 1. Then, the nodule level probability was converted into binary results using an optimal threshold determined by the receiver operating characteristic (ROC) curve. For the 3D CNN models, a nodule-level predictive probability of the high-risk factors can be directly obtained from the model. Similarly, we can receive a binary result using the same procedure described previously. In the prospective observational validation, the prespecified decision threshold of 0.40 (selected in the retrospective phase) was applied without modification to convert predicted probabilities to binary outputs. With the obtained predictive binary results, we can efficiently compute the metrics of sensitivity, specificity, PPV, NPV, and F1 according to their definitions. Additionally, the AUC was computed as the area under the ROC curve. All confidence intervals for AUC values were computed using bootstrapping. The Cohen’s kappa values were also computed. All of the above calculations were computed using Python 3.8.

### Reporting guideline adherence

This study has been reported in accordance with the STROCSS (Strengthening the Reporting of Cohort, Cross-sectional and Case-Control Studies in Surgery) criteria^[^51^]^.

## Results

### Summary statistics of the development cohorts

The A Cohort – A total of 268 patients (104 men, 164 women, mean age of 60.29 ± 9.69 years) who underwent surgery in Beijing Chao-Yang Hospital were included to develop the KB-GCN model. There were a total of 297 pulmonary nodules among the included 268 patients, all of which were diagnosed with early-stage lung adenocarcinoma. Among the 297 pulmonary nodules, 105 were confirmed to have high-risk factors by reviewing the pathology sections, while the rest were not. All confirmed nodules were annotated with their specific locations (i.e., *X, Y*, and *Z* coordinates). For the proposed KB-GCN model, one sample unit is a one-slice CT image. Therefore, for each pulmonary nodule, we only need to include all slices that cover it. We then randomly divide the whole cohort into two sets with a proportion of 7:3, in which 70% of the data used to train the model, and 30% of the data used to evaluate and select the best model. In summary, we obtained a total of 7330 slices, with 5600 in the training set and 1730 in the validation set for internal validation.

The B Cohort – A total of 68 patients (35 men, 33 women, mean age of 60.59 ± 10.00 years) who underwent surgery in the Third Xiangya Hospital were included as an independent external cohort to validate the proposed model. All the patients were diagnosed with early-stage lung adenocarcinoma. There were a total of 75 pulmonary nodules, of which 34 were identified with high-risk factors. For the B cohort, we obtained a total of 1827 slice for independent validation. The clinical and demographic information for the B and A cohorts is presented in Supplemental Digital Content Table 2, available at: http://links.lww.com/JS9/H79.

### Model performance

For the internal validation cohort (i.e., A cohort), the proposed KB-GCN model achieved an AUC of 0.92 (95% CI: 0.86 to 0.97). To determine an operational decision threshold, we constructed a ROC curve using the internal validation subset of the A cohort. The optimal cut-off value was set at 0.40, corresponding to the maximum Youden index (sensitivity + specificity − 1). Because sensitivity was prioritized for clinical safety (reducing missed PHRF-positive cases), a slightly sensitivity-favored threshold near the ROC optimum (0.40) was selected rather than the point of equal sensitivity and specificity. The proposed model achieved a sensitivity, specificity, positive predictive value (PPV), negative predictive value (NPV), and F1 score of 0.85, 0.92, 0.85, 0.92, and 0.85, respectively. The AUC value of the external independent B cohort is 0.88 (95% CI: 0.81 to 0.94). With an optimal cut-off value of 0.40 determined by the ROC curve, the proposed model achieved a sensitivity, specificity, PPV, NPV, and F1 score of 0.76, 0.80, 0.76, 0.80, and 0.76, respectively. Supplemental Digital Content Table 3, available at: http://links.lww.com/JS9/H79, shows the detailed metric results for both cohorts.

For the comparison study, we present only the results obtained on the external B cohort for simplicity. We report the results of six CNN models in Supplemental Digital Content Table 1, available at: http://links.lww.com/JS9/H79. They are 2D VGG16, 2D Inception V3, 2D ResNet50, 3D VGG16, 3D Inception V3, and 3D ResNet50, respectively. 3D VGG16 achieved the highest AUC (0.79) among the CNN baselines, which is lower than the proposed GCN model. This indicates that the proposed KB-GCN model outperforms all of the classical CNN-type models. This further demonstrates the effectiveness of using spatially related information with the GCN model.

### Summary statistics of the prospective validation cohort

The prospective cohort comprised 200 patients (63 males; median age 63 years) with early-stage lung adenocarcinoma, divided into PHRF-positive (*n* = 96, 48.0%) and PHRF-negative (*n* = 104, 52.0%) groups. Clinically significant disparities (*P* < 0.05) were observed between groups: PHRF-positive tumors were associated with higher male prevalence (41.67% vs 22.12%), smoking history (current/former smokers: 26.04% vs 11.54%), and predominantly part-solid nodule morphology (75.0% vs 38.46%). Surgically, PHRF-positive patients more frequently underwent lobectomy (80.21% vs 47.12%), correlating with advanced T-stage (T1c: 41.67% vs 14.42%) and nodal metastasis (N1/N2: 22.92% vs 0%). IASLC grading revealed marked biological aggressiveness in PHRF-positive tumors, with 55.21% classified as Grade 3, compared with 0.96% in the PHRF-negative group. Supplemental Digital Content Table 4, available at: http://links.lww.com/JS9/H79 summarizes the baseline characteristics of patients with and without PHRFs in the prospective cohort.

### Results of the prospective validation cohort

**Figure 4.**
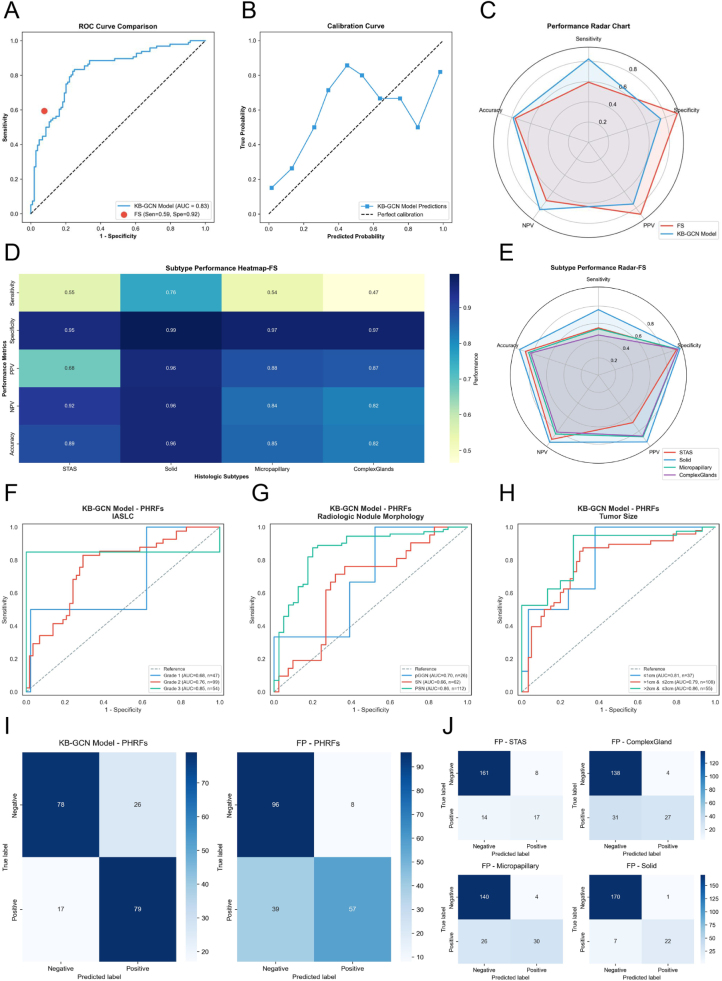
Comparative performance evaluation of the KB-GCN Model vs FS in PHRFs Prediction. (A) ROC curves comparing the KB-GCN model (blue line, AUC = 0.83) and FS (red dot, Sensitivity = 59%, specificity = 92%). (B) Calibration plot for the KB-GCN model (blue line) against perfect calibration (dashed line). (C) Radar chart comparing KB-GCN model(blue) and FS (red) across sensitivity, specificity, NPV, PPV, and F1 score. (D–E) Heatmap (D) and radar chart (E) illustrating performance of FS across different PHRFs’ subtypes (solid, micropapillary, complex glandular architecture, STAS). (F) ROC curves for KB-GCN model across IASLC grades (Grade 1: AUC: 0.68; Grade 2: AUC: 0.76; Grade 3: AUC: 0.85). (G) KB-GCN model performance by nodule type: part-solid nodules (PSN, AUC: 0.86) outperform pure ground-glass (pGGN, AUC: 0.70) and solid nodules (SN, AUC: 0.66). H. Receiver operating characteristic curves of KB-GCN Model by tumor size in the prospective cohort: ≤1 cm (*n* = 37, AUC = 0.815), >1 to ≤2 cm (*n* = 108, AUC = 0.785), and >2 to ≤3 cm (*n* = 55, AUC = 0.860). I. Confusion Matrices (KB-GCN vs FS), Left: The KB-GCN Model (T*N* = 78, F*P* = 26, F*N* = 17, T*P* = 79); Right: FS (T*N* = 96, F*P* = 8, F*N* = 39, T*P* = 57). (J) Four confusion matrices for FS across different PHRFs’ subtypes (solid, micropapillary, complex glandular architecture, and STAS).

In intraoperative prediction, FS assessment achieved moderate overall accuracy (77%) but exhibited critical limitations in sensitivity (59% for PHRF-positive cases), missing 40.62% (39/96) of high-risk cases (Table 1, Fig. 4I). The KB-GCN model significantly improved sensitivity to 82% (+23% absolute increase) with an AUC of 0.83 (Fig. 4A), demonstrating robust discrimination in ROC analysis. Calibration curves (Fig. 4B) further confirmed its reliability, with predicted probabilities closely aligned to actual PHRF-positive prevalence across risk strata. Radar plot comparisons (Fig. 4C) highlighted the model’s balanced performance: while FS excelled in specificity (92% vs 75%), the KB-GCN model surpassed FS in sensitivity (+23%), NPV (+11%), and F1 score (+8%).

**Table 1:** Detailed metric results for the Prospective Cohort.

Datasets	Accuracy	Sensitivity	Specificity	PPV	NPV	F1 Score	AUC
FS Overall	0.77	0.59	0.92	0.88	0.71	0.71	-
FS-STAS	0.89	0.55	0.95	0.68	0.92	0.61	-
FS-Solid	0.96	0.76	0.99	0.96	0.96	0.85	-
FS-Micropapillary	0.85	0.54	0.97	0.88	0.84	0.67	-
FS-Complex Glands	0.83	0.47	0.97	0.87	0.82	0.61	-
KB-GCN Model Overall	0.79	0.82	0.75	0.75	0.82	0.79	0.83

Notably, FS showed extreme variability across histologic subtypes (Fig. 4D and E): despite high specificity (>95% for all subtypes), its sensitivity ranged from 47% (complex glandular architecture) to 76% (solid), with STAS detection being particularly unreliable (sensitivity 55%, F1 61%). In contrast, the KB-GCN model demonstrated gradient discrimination across tumor biological aggressiveness, with AUC increasing from 0.68 (Grade 1) to 0.85 (Grade 3) (Fig. 4F). This aligns with the IASLC grading criteria, in which high-grade tumors (Grade 3) exhibit more distinct CT morphologic signatures, such as spiculation and heterogeneous enhancement. In nodule morphology subgroups (Fig. 4G), the model demonstrated superior performance in part-solid nodules (PSN; AUC: 0.86) compared to pure ground-glass nodules (pGGN; AUC: 0.70) or solid nodules (SN; AUC: 0.66). This performance advantage in PSNs is likely attributable to the inherent contrast between ground-glass and solid components, which provides richer discriminative features for the model. Within the PSN subgroup, the KB-GCN model achieved the highest sensitivity (87.5%), precision (87.5%), and F1 score (87.5%) among all morphological categories (Supplemental Digital Content Figure 2K, available at: http://links.lww.com/JS9/H78), consistent with its highest observed AUC (0.86, Fig. 4G). Specificity in the PSN subgroup was 77.5%. Compared to intraoperative FS, the KB-GCN model yielded significantly higher sensitivity (87.5% vs 68.06%) for detecting PHRFs in PSNs. Conversely, FS demonstrated higher specificity (92.5% vs 77.5%; Supplemental Digital Content Figure 2H, available at: http://links.lww.com/JS9/H78). Tumor size stratification (Fig. 4H) showed the highest discrimination in 2–3 cm lesions (AUC: 0.86, *n* = 55), followed by ≤1 cm (AUC: 0.815, *n* = 37) and 1–2 cm (AUC: 0.785, *n* = 108). Consistent with the confusion-matrix analysis (Supplemental Digital Content Figure 2, available at: http://links.lww.com/JS9/H78), FS outperformed KB-GCN model in ≤1 cm lesions (Accuracy 91.9% vs 81.1%; F1 76.9% vs 53.3%; Sensitivity 62.5% vs 50.0%), whereas the KB-GCN model surpassed FS in 1–2 cm (F1 75.9% vs 62.2%; Sensitivity 85.4% vs 47.9%) and 2–3 cm tumors (Accuracy 81.8% vs 70.9%; F1 87.2% vs 78.4%; Sensitivity 85.0% vs 72.5%). Supplemental Digital Content Table 5, available at: http://links.lww.com/JS9/H79, provides detailed insights into the clinical characteristics of cases where discrepancies exist among the KB-GCN model, FS, and FP assessments. Comparative Confusion Matrices of FP and KB-GCN models stratified by Radiologic Morphology, IASLC Grade, and Tumor Size Groups are shown in Supplemental Digital Content Figure 2, available at: http://links.lww.com/JS9/H78.

Interpretation of discordant cases (Table 2). Among false judgments with positive FP, FS false negatives (FP+ and FS−, *n* = 39) were mainly due to missed high-grade components: micropapillary >5% in 61.5% (24/39) and complex glandular architecture >5% in 41.0% (16/39). STAS positivity was less frequent (15.4%, 6/39), and solid components >5% were rare (7.7%, 3/39). In contrast, KB-GCN model false negatives (FP+ and KB-GCN−, *n* = 17) were likewise driven by micropapillary >5% (70.6%, 12/17) and complex glandular architecture >5% (64.7%, 11/17), but had higher proportions of STAS (35.3%, 6/17) and solid components >5% (29.4%, 5/17). Among false judgments with negative FP, FS false positives (FP− and FS+, *n* = 8) mainly arose from STAS (62.5%, 5/8) and complex glandular architecture (50.0%, 4/8), whereas the KB-GCN model false positives (FP− and KB-GCN+, *n* = 26) lacked permanent-pathology support but exhibited invasive CT signs (for example, GGO-solid interfaces within PSNs, textural heterogeneity, spiculation/lobulation, vessel convergence, pleural signs). Percentages may exceed 100% because components can coexist.

**Table 2:** Pathological characteristics of discrepant cases among the KB-GCN model, frozen section and final pathology.

	FP(-)				FP(+)			
Characteristics	FS(-)	FS(+)	KB-GCN Model(-)	KB-GCN Model(+)	FS(-)	FS(+)	KB-GCN Model(-)	KB-GCN Model(+)
KB-GCN Model								
HRFs (-)	73/96 (76.04%)	5/8 (62.50%)	78/78 (100.00%)	-	6/39 (15.38%)	11/57 (19.30%)	17/17 (100.00%)	-
HRFs (+)	23/96 (23.96%)	3/8 (37.50%)	-	26/26 (100.00%)	33/39 (84.62%)	46/57 (80.70%)	-	79/79 (100.00%)
Frozen Sections (FS)								
HRFs (-)	96/96 (100.00%)	-	73/78 (93.59%)	23/26 (88.46%)	39/39 (100.00%)	-	6/17 (35.29%)	33/79 (41.77%)
HRFs (+)	-	8/8 (100.00%)	5/78 (6.41%)	3/26 (11.54%)	-	57/57 (100.00%)	11/17 (64.71%)	46/79 (58.23%)
STAS (-)	96/96 (100.00%)	3/8 (37.50%)	75/78 (96.15%)	24/26 (92.31%)	39/39 (100.00%)	37/57 (64.91%)	14/17 (82.35%)	62/79 (78.48%)
STAS (+)	-	5/8 (62.50%)	3/78 (3.85%)	2/26 (7.69%)	-	20/57 (35.09%)	3/17 (17.65%)	17/79 (21.52%)
Solid ≥ 5%(-)	96/96 (100.00%)	8/8 (100.00%)	78/78 (100.00%)	26/26 (100.00%)	39/39 (100.00%)	34/57 (59.65%)	15/17 (88.24%)	58/79 (73.42%)
Solid ≥ 5%(+)	-	-	-	-	-	23/57 (40.35%)	2/17 (11.76%)	21/79 (26.58%)
Micropapillary ≥ 5%(-)	96/96 (100.00%)	5/8 (62.50%)	76/78 (97.44%)	25/26 (96.15%)	39/39 (100.00%)	26/57 (45.61%)	11/17 (64.71%)	54/79 (68.35%)
Micropapillary ≥ 5%(+)	-	3/8 (37.50%)	2/78 (2.56%)	1/26 (3.85%)	-	31/57 (54.39%)	6/17 (35.29%)	25/79 (31.65%)
Complex glands ≥ 5%(-)	96/96 (100.00%)	4/8 (50.00%)	76/78 (97.44%)	24/26 (92.31%)	39/39 (100.00%)	30/57 (52.63%)	13/17 (76.47%)	56/79 (70.89%)
Complex glands ≥ 5%(+)	-	4/8 (50.00%)	2/78 (2.56%)	2/26 (7.69%)	-	27/57 (47.37%)	4/17 (23.53%)	23/79 (29.11%)
Final Pathology (FP)								
HRFs (-)	96/96 (100.00%)	8/8 (100.00%)	78/78 (100.00%)	26/26 (100.00%)	-	-	-	-
HRFs (+)	-	-	-	-	39/39 (100.00%)	57/57 (100.00%)	17/17 (100.00%)	79/79 (100.00%)
STAS (-)	96/96 (100.00%)	8/8 (100.00%)	78/78 (100.00%)	26/26 (100.00%)	33/39 (84.62%)	32/57 (56.14%)	11/17 (64.71%)	54/79 (68.35%)
STAS (+)	-	-	-	-	6/39 (15.38%)	25/57 (43.86%)	6/17 (35.29%)	25/79 (31.65%)
Solid ≥ 5%(-)	96/96 (100.00%)	8/8 (100.00%)	78/78 (100.00%)	26/26 (100.00%)	36/39 (92.31%)	31/57 (54.39%)	12/17 (70.59%)	55/79 (69.62%)
Solid ≥ 5%(+)	-	-	-	-	3/39 (7.69%)	26/57 (45.61%)	5/17 (29.41%)	24/79 (30.38%)
Micropapillary ≥ 5%(-)	96/96 (100.00%)	8/8 (100.00%)	78/78 (100.00%)	26/26 (100.00%)	15/39 (38.46%)	25/57 (43.86%)	5/17 (29.41%)	35/79 (44.30%)
Micropapillary ≥ 5%(+)	-	-	-	-	24/39 (61.54%)	32/57 (56.14%)	12/17 (70.59%)	44/79 (55.70%)
Complex glands ≥ 5%(-)	96/96 (100.00%)	8/8 (100.00%)	78/78 (100.00%)	26/26 (100.00%)	23/39 (58.97%)	15/57 (26.32%)	6/17 (35.29%)	32/79 (40.51%)
Complex glands ≥ 5%(+)	-	-	-	-	16/39 (41.03%)	42/57 (73.68%)	11/17 (64.71%)	47/79 (59.49%)

HRFs = pathological high-risk factors; STAS = Spread Through Air Spaces.

Data represent the number of patients, with percentages (%) in parentheses.

Decision-curve analysis (DCA; Supplemental Digital Content Figure 4, available at: http://links.lww.com/JS9/H78) quantified clinical utility across threshold probabilities in the prospective cohort (*n* = 200; PHRF prevalence 52%). Both the KB-GCN model and FS achieved positive net benefits relative to Treat-None across wide ranges. FS provided higher net benefit at low-to-mid thresholds (0.13–0.45). The KB-GCN model surpassed FS, increasing from 0.46 to 0.82, and remained beneficial up to ~0.83. At clinically relevant thresholds, the advantage of the KB-GCN model was substantial: at 0.60, the net benefit was 0.2625 for the KB-GCN model versus 0.1875 for FS (≈7.5 more net-beneficial decisions per 100 patients); at 0.70, 0.1917 versus 0.0250 (≈16.7 per 100). Treat-All dominated only at very low thresholds (< ~ 0.12), while Treat-None dominated at very high thresholds (≥ ~0.83). These findings indicate that the KB-GCN model offers greater overall clinical benefit at moderate-to-high action thresholds where false-positive penalties are largest.

In the Prospective Cohort (*n* = 200; PHRF prevalence 48%), concordance between the KB-GCN and frozen section (FS) recommendations was 64.0% (κ = 0.292). Compared with the actual surgical procedure performed, the agreement was 61.5% for FS recommendations (κ = 0.294) and 68.5% for KB-GCN recommendations (κ = 0.362). Using the final pathology (FP)-guided optimal approach as the reference standard, the overall appropriateness of the recommended strategy was 78.5% for KB-GCN (157/200; κ = 0.571) and 76.5% for FS (153/200; κ = 0.523). The actual intraoperative approach agreed with the FP-guided optimal approach in 66.0% of cases (132/200; κ = 0.327). Analysis stratified by tumor size revealed that KB-GCN demonstrated higher agreement with the final actual surgery for tumors 1–2 cm (65.7%, κ = 0.293) and 2–3 cm (72.7%, κ = 0.252), whereas FS retained higher agreement for tumors ≤1 cm (75.7%, κ = 0.268). Based on the actual surgical approach, undertreatment (PHRF-positive cases receiving sublobar resection) occurred in 9.5% (19/200) and overtreatment (PHRF-negative cases receiving lobectomy) in 24.5% (49/200). Notably, among the 13 PHRF-positive cases that were FS-negative and underwent sublobar resection, KB-GCN correctly identified 9 (69.2%) as positive preoperatively, highlighting its potential to reduce undertreatment. A completion surgery triggered by FP within 90 days occurred in only 0.5% (1/200) of patients. In the clinically pivotal 1–3 cm tumor group, KB-GCN demonstrated superior agreement with the FP-guided standard and yielded more diagnostic upgrades that could have prevented undertreatment than FS, consistent with its higher sensitivity. For lesions ≤1 cm, FS demonstrated higher appropriateness and specificity, aligning with the known indolent biology of many small nodules.

## Discussion

This represents the first prospective observational study to compare the accuracy of intraoperative FS analysis with that of a preoperative CT-based deep learning model in assessing PHRFs in cT1N0M0 invasive lung adenocarcinoma. Since its introduction, intraoperative FS assessment has remained the standard method for differentiating indeterminate pulmonary lesions and identifying adenocarcinoma in situ (AIS), minimally invasive adenocarcinoma (MIA), and invasive adenocarcinoma, thereby guiding surgical decision-making^[^63^]^. However, previous investigations have demonstrated suboptimal sensitivity of intraoperative FS in detecting PHRFs^[^26,28,64^]^, thereby limiting its widespread clinical adoption. To address this critical gap, our team developed the KB-GCN model – a deep-learning system based on preoperative chest CT scans for PHRF identification – whose performance was satisfactory in both internal and external validation cohorts. Subsequent prospective validation revealed that the KB-GCN model significantly outperforms FS in sensitivity (82% vs 59%), but has lower specificity (75% vs 92%). These findings suggest that this CT-based artificial intelligence model could potentially serve as an auxiliary predictive tool for identifying early-stage lung cancer patients with PHRFs, providing crucial decision support for surgical teams and pathologists.

Current guidelines, informed by the JCOG0802 (UMIN000002317) and CALGB 140503 (NCT00499330) randomized controlled trials, suggest that sublobar resection demonstrates non-inferior oncological outcomes compared to lobectomy for non-small cell lung cancers (NSCLC) ≤2 cm^[^4,65^]^. However, these landmark studies did not fully account for the prognostic impact of PHRFs in invasive adenocarcinoma. Emerging evidence indicates that lobectomy remains preferable for tumors harboring PHRFs, particularly given their association with elevated lymph node metastasis risk^[^17,66,67^]^. Accurate preoperative identification of PHRFs is pivotal in shaping surgical planning, determining both the extent of parenchymal resection and the lymph node dissection strategy. Nevertheless, conventional intraoperative FS analysis exhibits critical limitations in this context. The FS’s suboptimal sensitivity (59% in our Prospective Cohort) may lead to undertreatment of peripheral PHRF-positive tumors through inappropriate sublobar resections. In the Prospective Cohort, 40.63% (39/96) of PHRF-positive tumors were misclassified by FS, potentially leading to inadequate sublobar resections in peripherally located high-risk lesions. Furthermore, the inherent workflow constraints of intraoperative FS pose challenges for operative planning. Given the absence of reliable preoperative evaluation for PHRFs, a staged surgical strategy – initial wedge resection for histopathological confirmation followed by completion lobectomy – remains the clinical standard in thoracic oncology practice. In the ideal surgical scenario, however, anatomically precise segmentectomy with meticulous preoperative planning achieves oncologically adequate resection margins for deep-seated pulmonary tumors devoid of PHRFs, preserving more lung tissue. In the Prospective Cohort, 7.69% (8/104) of ultimately PHRF-negative cases experienced unnecessary parenchymal loss due to false-positive FS results, leading to lobectomy. These inherent limitations of FS underscore the critical need for accurate preoperative assessments to counteract its shortcomings.

In this study, we introduced the KB-GCN model for preoperative prediction of PHRFs using chest CT imaging. This model is a graph convolutional neural network that leverages a novel knowledge-based approach to characterize the 3D spatial information of medical images. It better captures spatial information than conventional approaches that use 3D convolution kernels for feature extraction. Our prospective study shows the KB-GCN model achieves slightly higher accuracy than FS in predicting PHRFs. Although specificity decreases, detection sensitivity improves significantly. This advancement partially mitigates the limitations of intraoperative FS by enabling preoperative assessment of PHRFs in lung adenocarcinoma. This shift not only optimizes thoracic surgeons’ surgical planning but also provides potential guidance for pathologists during intraoperative FS assessment.

Methodological innovation relative to prior AI approaches. The KB-GCN model differs from conventional CNN- and radiomics-based pipelines in two key ways. First, it leverages a pretrained image encoder (VGG16) as a knowledge base to derive slice-level semantic representations that serve as node attributes. Second, it builds a slice graph in which edges encode axial spatial continuity and inter-slice proximity, allowing the GCN to propagate and aggregate information across slices. This relational modeling captures dependencies and heterogeneous composition within a nodule (e.g., ground-glass versus solid components) that are often ignored by 2D CNNs or diluted by monolithic 3D convolutions. By dispensing with hand-crafted radiomics features, the model learns hierarchical, data-driven representations while retaining interpretability through the explicit graph structure. These design choices plausibly explain the superior discrimination observed over six strong CNN baselines and the model’s particular strength in part-solid nodules and small tumors, where inter-slice heterogeneity and sampling constraints are most pronounced.

Our retrospective analysis demonstrated that the model performed consistently across CT scanners from different manufacturers, including SIEMENS, Philips, GE MEDICAL SYSTEMS, UIH, and NMS. The true positive rates and predicted positive rates were comparable across devices (e.g., SIEMENS: true positive rate 0.4444, predicted positive rate 0.4343; Philips: true positive rate 0.3333, predicted positive rate 0.3611; GE MEDICAL SYSTEMS: true positive rate 0.3385, predicted positive rate 0.3594), and a chi-square test revealed no statistically significant differences (*χ*^2^ = 1.6963, *P* = 0.4282). This suggests that the preprocessing steps effectively standardized the input data, mitigating the impact of variations in acquisition settings. Thus, the model shows promising generalizability for clinical application across diverse CT platforms.

Notably, although our KB-GCN model was intentionally designed as an imaging-only framework to evaluate the discriminative power of CT-derived features independent of clinical information, we observed that its predictions nonetheless captured key clinical risk patterns. Post-hoc analysis revealed that the model-predicted high-risk group showed significantly higher proportions of males (44.62% vs 25.19%, *χ*^2^ = 6.80, *P* = 0.009) and individuals with smoking history (27.69% vs 14.07%, *χ*^2^ = 4.53, *P* = 0.033) compared to the low-risk group in the prospective validation cohort. This distribution closely mirrored the actual PHRF + group demographics (41.67% males, 26.04% with smoking history). Furthermore, a graded relationship emerged in smoking status categories, with the highest proportion of current smokers concentrated in the predicted high-risk group (16.92% vs 6.67% in the low-risk group). These findings suggest that, despite the absence of explicit integration of clinical variables, the imaging-derived features learned by our model effectively capture biological patterns associated with established clinical risk factors, thereby enhancing the biological plausibility of its predictions. Future iterations could explore multimodal integration of clinical variables with imaging features to potentially further improve predictive accuracy.

Subgroup analysis revealed that for invasive lung adenocarcinoma presenting as pGGN, both the sensitivity and specificity of intraoperative FS exceeded those of the KB-GCN model – attributable primarily to the very low prevalence of PHRFs in pGGN. Given the favorable prognosis associated with pGGN, the clinical relevance of identifying PHRFs in such lesions is limited. In contrast, for pure SN and PSN, the sensitivity of intraoperative FS was significantly lower than that of the KB-GCN model (SN: 28.57% vs 71.43%; PSN: 68.06% vs 87.5%), though FS maintained higher specificity (SN: 90.24% vs 65.85%; PSN: 92.5% vs 77.5%). In PSN, the coexistence of ground-glass and solid components reflects lepidic and invasive histologic patterns, respectively, where the solid portion correlates strongly with PHRFs. This radiologic-pathologic correlation yields high-contrast CT features – such as abrupt attenuation gradients, heterogeneous texture, lobulation, spiculation, and pleural signs – that provide rich discriminative information. The KB-GCN model, designed to integrate relational intra- and peritumoral data along with the proportion of solid and ground-glass components, demonstrated optimal performance in PSN (AUC: 0.86; sensitivity 87.5%; specificity 77.5%), achieving higher sensitivity than FS with a moderate trade-off in specificity. Conversely, the model showed lower sensitivity in pGGN due to lesion homogeneity and low PHRF prevalence, and reduced specificity in SN, likely due to uniform attenuation. These findings underscore the model’s particular advantage in evaluating PSN.

When analyzed by tumor diameter, ≤1 cm lesions showed a distinct pattern (Supplemental Digital Content Figure 2, available at: http://links.lww.com/JS9/H78): intraoperative FS achieved high accuracy (91.89%) driven by perfect specificity (100.0%) but limited sensitivity (62.50%), whereas the KB-GCN model performed more modestly (AUC: 0.815; sensitivity 50.00%; accuracy 81.08%). This likely reflects inherent limits of CT for very small nodules – subtle spiculation, micro-cavitation, and fine textural heterogeneity are often under-resolved – constraining deep-learning detectability. By contrast, FS can frequently sample the entire lesion, allowing detection of focal high-risk areas that may be occult on CT.

For tumors 1–2 cm in diameter, a critical reversal was observed. FS exhibited its lowest sensitivity (47.92%) in determining PHRFs, despite maintaining high specificity (95.00%). the KB-GCN model’s high sensitivity in 1–2 cm tumors (85.42%) aligns with the premise that lesions of this size present more discernible imaging features indicative of aggressive behavior, such as irregular margins or heterogeneous attenuation. FS, however, suffers from increased sampling error because the impracticality of exhaustively evaluating the entire lesion intraoperatively leads to missed high-risk foci.

For 2–3 cm tumors, the KB-GCN model achieved balanced, superior discrimination (AUC: 0.86), outperforming FS on both sensitivity (85.0% vs 72.5%) and specificity (73.3% vs 66.7%). We attribute this to the richer and more stable radiographic heterogeneity and morphological cues in larger lesions – such as conspicuous borders, internal attenuation variability, and architectural complexity – that provide CT-based models with stronger signals of biological aggressiveness. In contrast, FS accuracy tends to decline as tumor size increases because intratumoral heterogeneity exacerbates sampling error; pathologists cannot exhaustively evaluate the entire lesion intraoperatively and may miss focal high-risk zones.

Our error analysis indicates that FS false negatives predominantly involved micropapillary and complex glandular architecture, likely reflecting limited intraoperative sampling of small, spatially heterogeneous high-grade foci and challenges in recognizing subtle patterns on FS. This is consistent with frozen-section literature on STAS pitfalls, where misdiagnoses have been attributed to: location-related factors including clusters near the section edge and near the main tumor edge, difficulty distinguishing suspicious clusters from non-neoplastic cells such as tumor-associated macrophages, artifact clusters specific to FS, and rare isolated clusters lacking contiguous “trails”^[^64^]^. In contrast, the KB-GCN model, whose false negatives were also enriched for micropapillary/complex glandular architecture, showed higher proportions of missed STAS and focal solid components, underscoring the limited CT visibility of micro-scale or low-contrast features. FS false positives were often due to overcalling STAS and complex glandular architecture, potentially related to freezing artifacts and misinterpretation of alveolar spaces, whereas the KB-GCN model false positives reflected invasive-appearing imaging phenotypes without permanent-pathology support. Collectively, these complementary limitations support a dual-channel strategy – preoperative CT-based AI plus intraoperative FS – to improve overall detection of high-risk histologic features in 1–3 cm lesions with solid components.

Although the KB-GCN model has lower specificity than FS, DCA showed that the model yields higher net clinical benefit across moderate-to-high thresholds (0.46–0.82), where the cost of overtreatment is most strongly penalized. Importantly, our prospective validation was observational: the KB-GCN model outputs were not used to change the operative plan. All cases followed intraoperative FS and prevailing standards. Consequently, the KB-GCN model false positives (*n* = 26) did not cause incremental parenchymal loss or model-attributable complications. In contrast, FS false positives (*n* = 8) contributed to unnecessary parenchymal loss within the staged wedge-then-completion workflow. These results support a dual-channel strategy – preoperative KB-GCN model plus intraoperative FS – to improve overall detection while managing the risk of overtreatment. Therefore, it is necessary to conduct new research to integrate the KB-GCN model into the diagnosis and treatment decision-making process. PHRFs largely reflect the biological aggressiveness of invasive adenocarcinoma. Currently, sublobar resection is typically applied to tumors less than 3 cm. Therefore, accurate determination of PHRFs in lesions with solid components (diameter 1–3 cm) is critical for guiding surgical strategy. Through this study, we observed that artificial intelligence can serve as an expert companion to thoracic surgeons and pathologists. In the future, combining preoperative AI model-assisted evaluation with intraoperative FS verification could facilitate more accurate and consistent PHRFs assessment.

Our findings indicate that KB-GCN recommendations demonstrated higher concordance with the ultimate surgical approach than FS recommendations (68.5% vs 61.5%), despite the surgical team being blinded to the AI output. This suggests that the model’s preoperative risk stratification captures clinically relevant information that aligns with final surgical decisions. A key observation was that among PHRF-positive cases, which were FS-negative and consequently underwent sublobar resection, KB-GCN would have correctly identified 69.2% (9/13) preoperatively. This highlights its potential utility in a prospective setting to mitigate undertreatment. These results, supported by decision-curve analyses, advocate a dual-channel diagnostic workflow in which preoperative KB-GCN risk assessment complements intraoperative FS, particularly for clinically pivotal 1–3 cm tumors with solid components. It is crucial to interpret these findings in light of the observational nature of this study. The “actual surgery” was a complex decision informed by intraoperative FS, margin assessment, anatomy, patient comorbidities, and surgeon judgment. Therefore, agreement with the actual procedure should not be misconstrued as an endorsement of AI guidance over clinical acumen. Instead, our analyses quantify the potential complementarity between preoperative AI and intraoperative FS and identify specific tumor size strata where each modality demonstrates relative strength. This groundwork necessitates prospective interventional trials to validate defined algorithmic pathways – such as rules for management when AI and FS are concordant and strategies for resolving discordant results. Such studies are essential to directly measure the impact of AI integration on critical outcomes, including model-attributable rates of overtreatment and undertreatment, parenchymal preservation, margin status, lymph node assessment strategy, and complication profiles.

**Figure 5.**
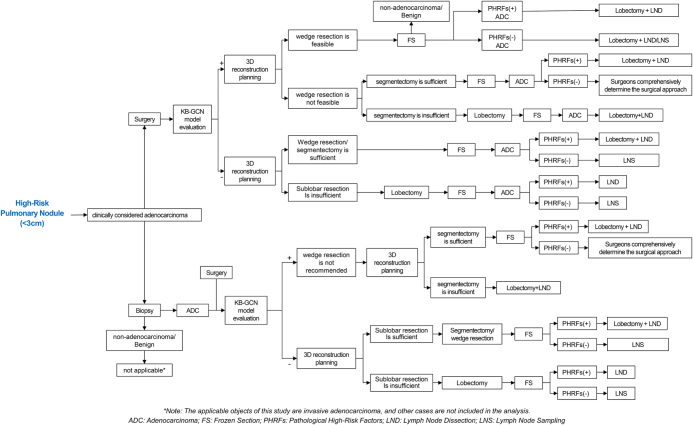
Flowchart of the potential clinical application for the KB-GCN model. The workflow illustrates how the preoperative risk stratification by the KB-GCN model, based on CT imaging, can complement intraoperative frozen section (FS) analysis to guide surgical extent.

The ultimate goal of our KB-GCN model is its seamless integration into the clinical pathway to optimize surgical decision-making for early-stage LUAD. As illustrated in Figure 5, the model provides preoperative risk stratification that informs surgical planning. For patients classified as low-risk by the KB-GCN model, the surgical team can proceed with sublobar resection with greater confidence, especially when intraoperative FS findings are concordant. Conversely, a high-risk prediction should raise suspicion for aggressive disease. In such cases, even with an initially negative or inconclusive FS, the team may justifiably lean toward a more extensive resection (e.g., lobectomy) or perform more meticulous lymph node assessment – thereby leveraging the model’s high sensitivity to compensate for the inherent limitations of FS. Notably, our initial focus on maximizing sensitivity (82%) aligned with the unmet clinical need to minimize false negatives, which could otherwise lead to under-treatment and higher recurrence risks, although this came at the cost of a slight decrease in specificity. To mitigate this limitation, we introduced the clinical workflow in Figure 5, which enhances surgical accuracy by combining the preoperative KB-GCN model assessment with intraoperative FS findings. It is important to emphasize that the decision threshold of 0.40 used in this validation was derived from retrospective ROC analysis; the optimal threshold for clinical deployment may ultimately aim to maximize overall clinical benefit – balancing the avoidance of unnecessary lobectomies against inadequate sublobar resections. A critical future direction is to develop a dynamic fusion model that quantitatively integrates the preoperative KB-GCN model’s probability with intraoperative FS results to generate a composite risk score. This would enable context-aware threshold adjustment and further refine surgical guidance, supporting a more robust and standardized preoperative–intraoperative evaluation protocol.

Some limitations of our study are present. Our prospective validation was conducted at a single center. Although the model was developed using data from two institutions and showed intense discrimination in an independent external cohort (internal AUC: 0.92; external AUC: 0.88), a single-center prospective design constrains generalizability. Differences across institutions in CT hardware, reconstruction kernels, and slice thickness, contrast protocols, patient case-mix, and, critically, intraoperative FS preparation and interpretation workflows can alter both the imaging feature distribution and the stability of the reference standard. These “domain shift” factors may affect calibration and the applicability of the fixed operating threshold (0.40). To mitigate these risks, we prospectively locked the model architecture, weights, preprocessing, and decision threshold; standardized thin-slice CT inputs (0.5–1.5 mm) with resampling and intensity normalization; and reported performance stratified by nodule morphology, tumor size, and IASLC grade. However, rigorous multicenter prospective validation remains essential to establish real-world generalizability. Given documented inter-institutional variability in FS performance^[^64,68,69^]^, we have initiated registration and preparations for a multicenter prospective study to evaluate discrimination (AUC), calibration (e.g., slope/intercept), and clinical utility (e.g., decision curve analysis) across diverse scanners and workflows, and to develop a standardized preoperative AI plus intraoperative FS pathway. We will also assess site-specific probability calibration or threshold adaptation, where necessary, to ensure safe and reliable cross-institutional deployment. This study cohort consisted of East Asian participants, which reduces population stratification bias but limits generalizability. External validation in multiethnic, multicenter international cohorts is urgently needed to assess generalizability and to explore population-specific adjustments. Because the prospective validation was observational and the clinical team remained blinded to the KB-GCN model’s results, surgical decisions were not influenced by its predictions; we could not measure model-attributable overtreatment (incremental parenchymal loss or complications) in false-positive cases. The DCA provides the expected net benefit if the model were used, but real-world impact requires a trial where the KB-GCN model prospectively guides surgical extent.

Scope of PHRFs. We focused our primary endpoint on >5% high-grade components and STAS to enable a pragmatic intraoperative comparison with FS. VPI and LVI, although clinically meaningful, generally require special stains (e.g., elastin for VPI; D2-40/CD31/CD34 for LVI) and cannot be reliably assessed within the intraoperative window. STAS can be attempted on FS but is hampered by low sensitivity and freezing artifacts. To enable a rigorous and executable prospective comparison that reflects real-world intraoperative workflows, we therefore focused the primary endpoint on PHRFs that can be attempted by FS (>5% high-grade components and STAS). VPI and LVI were recorded on permanent sections when available, but were not included as primary endpoints. We acknowledge that excluding VPI/LVI may limit generalizability; accordingly, future work will extend the KB-GCN model to predict VPI/LVI using permanent pathology ground truth in larger multicenter cohorts and will evaluate integrated preoperative-intraoperative workflows. Second, the sample size used for model development was relatively limited. In fact, we attempted to expand the sample size during modeling, but this did not significantly enhance model performance. Future development of advanced deep learning algorithms through multicenter, large-scale training is necessary to address these issues. Finally, this study has not yet integrated preoperative deep learning evaluation with intraoperative FS assessment into a standardized workflow for PHRFs identification in invasive lung adenocarcinoma. This constitutes a novel research question requiring a dedicated prospective study to develop more efficient and accurate evaluation protocols.

In conclusion, this prospective study compared the accuracy of our KB-GCN model with that of FS in evaluating PHRFs. The results demonstrated that the KB-GCN model achieves superior sensitivity, effectively compensating for the limitations of intraoperative FS in diagnosing PHRFs for early-stage invasive lung adenocarcinoma, particularly in tumors (1–3 cm) with solid components. A critical future direction is to develop a dynamic fusion model that quantitatively integrates the preoperative KB-GCN probability with intraoperative FS results to generate a composite risk score. This would enable context-aware threshold adjustment and further refine surgical guidance, supporting a more robust and standardized preoperative–intraoperative evaluation protocol.

## Conclusions

This prospective study demonstrates that a preoperative deep learning model (KB-GCN model) based on CT scans significantly improves sensitivity for detecting PHRFs in early-stage lung adenocarcinoma compared to intraoperative FS. The KB-GCN model achieved 82% sensitivity (vs FS: 59%), misclassifying 23% fewer high-risk tumors than FS (which misclassified 40.6%), despite slightly lower specificity (75% vs 92%).

The KB-GCN model demonstrated strong performance in part-solid nodules (AUC: 0.86) and 2–3 cm tumors (AUC: 0.86), with moderate performance in ≤1 cm (AUC: 0.815) and 1–2 cm lesions (AUC: 0.785). In 1–3 cm tumors, the model consistently improved sensitivity and F1 over FS, supporting its use to optimize intraoperative decision-making.

These findings support the KB-GCN model as a potential preoperative decision-support tool. While the present study focused on PHRFs that are assessable intraoperatively (>5% high-grade components and STAS), future multicenter development will extend the KB-GCN model to VPI/LVI using permanent pathology ground truth, thereby broadening clinical applicability. It enables more informed surgical planning (lobectomy vs sublobar resection) and helps mitigate risks associated with the sensitivity limitations of intraoperative FS assessment. Future work should integrate this preoperative AI assessment with intraoperative FS into standardized clinical workflows.

## Data Availability

1. Data Restrictions: The retrospective (Cohorts A & B) and prospective cohort datasets used to develop and validate the KB-GCN model are not publicly available due to patient privacy regulations and institutional restrictions. 2. Controlled Access: These datasets may be made available for collaborative academic research upon reasonable request to the corresponding author (Dr. Ying Ji), subject to: Formal review by institutional ethics review boards. Execution of data use/confidentiality agreements. 3. Model & Supplemental Details: The full KB-GCN model architecture, training parameters, and validation metrics are provided in the Supplementary Materials. 4. Subgroup Analysis Data: Anonymized data underlying subgroup analyses will be shared publicly via the Chinese Clinical Trial Registry (ChiCTR2300073455) upon publication.

## Research registration unique identifying number (UIN)

This study was registered in the Chinese Clinical Trial Registry (registration number: ChiCTR2300073455) by investigators from Capital Medical University Affiliated Beijing Chaoyang Hospital, and detailed trial information can be accessed at the registry website https://www.chictr.org.cn/showproj.html?proj=196489.
